# (Nitrato-κ*O*)tris­[tris­(4-fluoro­phen­yl)phosphane-κ*P*]copper(I)

**DOI:** 10.1107/S1600536812043346

**Published:** 2012-10-24

**Authors:** Tania N. Hill, Andreas Roodt

**Affiliations:** a Department of Chemistry, University of the Free State, PO Box 339, Bloemfontein 9300, South Africa

## Abstract

In the title complex, [Cu(NO_3_)(C_18_H_12_F_3_P)_3_], the ligating atoms define a distorted tetrahedon with the three tris­(4-fluoro­phen­yl)phosphane ligands in the basal positions and the nitrate ligand in the axial position. The intra­molecular π–π inter­action [centroid–centroid distance = 3.6113 (11) Å] between two of the 4-fluoro­phenyl groups is complemented by both C—H⋯F and C—H⋯O inter­actions with distances in the range 2.51–2.60 Å, resulting in a tight head-to-tail packing.

## Related literature

For related complexes, see: Hanna *et al.* (2005 ▶); Steyl (2009 ▶); Saravanabharathi *et al.* (2002 ▶); Dyason *et al.* (1986 ▶); Matthew *et al.* (1971 ▶). 
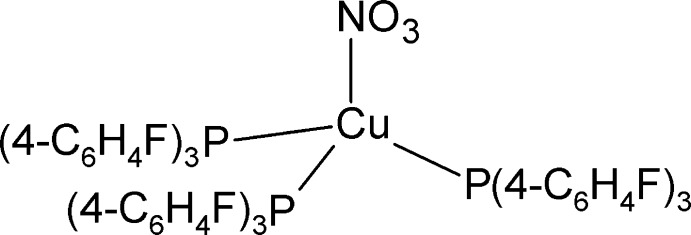


## Experimental

### 

#### Crystal data

[Cu(NO_3_)(C_18_H_12_F_3_P)_3_]
*M* *_r_* = 1074.29
Triclinic, 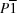

*a* = 9.3861 (3) Å
*b* = 12.2552 (4) Å
*c* = 21.4820 (7) Å
α = 85.274 (2)°
β = 86.843 (1)°
γ = 74.954 (1)°
*V* = 2376.76 (13) Å^3^
*Z* = 2
Mo *K*α radiation
μ = 0.64 mm^−1^
*T* = 100 K
0.38 × 0.11 × 0.08 mm

#### Data collection

Bruker X8 APEXII 4K KappaCCD diffractometer
Absorption correction: multi-scan (*SADABS*; Bruker, 2004 ▶) *T*_min_ = 0.792, *T*_max_ = 0.950
28458 measured reflections
11760 independent reflections
9439 reflections with *I* > 2σ(*I*)
*R*_int_ = 0.030

#### Refinement

*R*[*F* ^2^ > 2σ(*F* ^2^)] = 0.035
*wR*(*F* ^2^) = 0.085
*S* = 1.05
11760 reflections
640 parameters
H-atom parameters constrained
Δρ_max_ = 0.40 e Å^−3^
Δρ_min_ = −0.41 e Å^−3^

### 

Data collection: *APEX2* (Bruker, 2005 ▶); cell refinement: *SAINT-Plus* (Bruker, 2004 ▶); data reduction: *SAINT-Plus* and *XPREP* (Bruker, 2004 ▶); program(s) used to solve structure: *SHELXS97* (Sheldrick, 2008 ▶); program(s) used to refine structure: *SHELXL97* (Sheldrick, 2008 ▶); molecular graphics: *DIAMOND* (Brandenburg & Putz, 2005 ▶); software used to prepare material for publication: *WinGX* (Farrugia, 1999 ▶).

## Supplementary Material

Crystal structure: contains datablock(s) global, I. DOI: 10.1107/S1600536812043346/mw2090sup1.cif

Structure factors: contains datablock(s) I. DOI: 10.1107/S1600536812043346/mw2090Isup2.hkl

Additional supplementary materials:  crystallographic information; 3D view; checkCIF report

## Figures and Tables

**Table 1:** Selected bond lengths (Å)

O1—Cu1	2.1182 (12)
P1—Cu1	2.2901 (5)
P2—Cu1	2.2840 (5)
P3—Cu1	2.3256 (5)

**Table 2:** Hydrogen-bond geometry (Å, °)

*D*—H⋯*A*	*D*—H	H⋯*A*	*D*⋯*A*	*D*—H⋯*A*
C122—H122⋯O2	0.95	2.30	3.224 (2)	163
C336—H336⋯O1	0.95	2.17	3.037 (2)	151
C126—H126⋯F22^i^	0.95	2.40	3.281 (2)	154
C136—H136⋯O3^ii^	0.95	2.53	3.223 (2)	130
C215—H215⋯F13^iii^	0.95	2.51	3.301 (2)	141
C315—H315⋯F33^iv^	0.95	2.50	3.403 (2)	159
C332—H332⋯F32^v^	0.95	2.48	3.131 (2)	125
C326—H326⋯F33^vi^	0.95	2.36	3.150 (2)	141

Symmetry codes: (i) 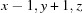; (ii) 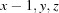; (iii) 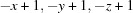; (iv) 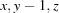; (v) 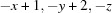; (vi) 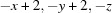.

**Fig. 1.**
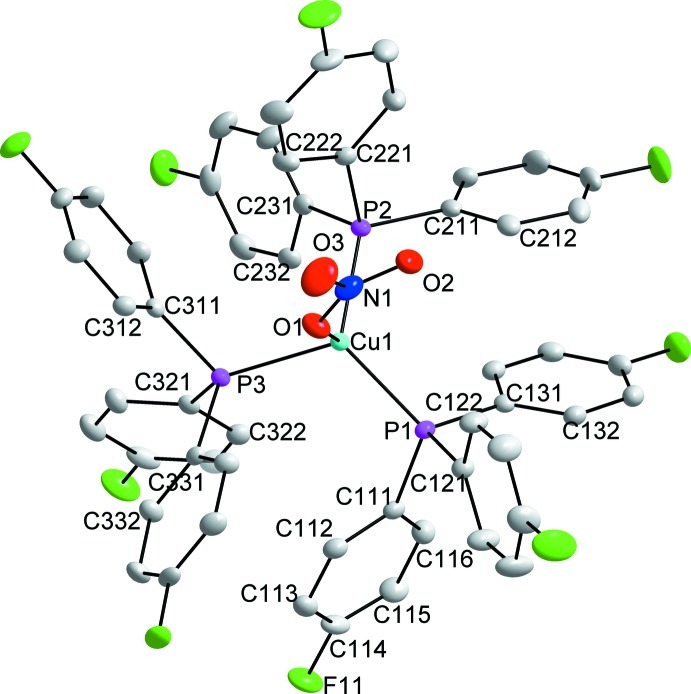
View of I (50% probability displacement ellipsoids). Phenyl rings are numbered Cxyz where x represents the phosphane to which the ring is attached, y represents the ring number and z the atom number in the ring. Only the first phenyl ring is completely numbered for illustrative purpose Hydrogen atoms have been omitted for clarity.

**Fig. 2.**
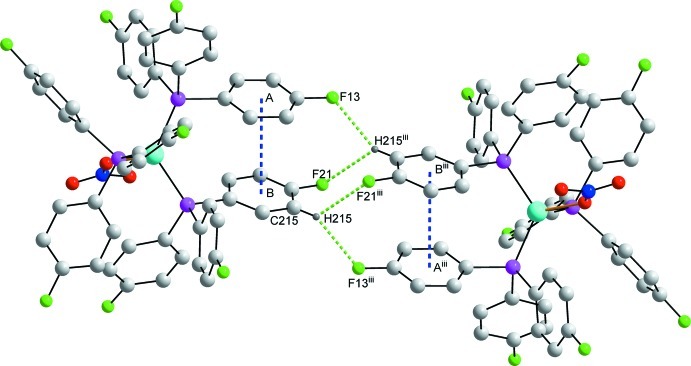
Intermolecular C—H···F and intramolecular π–π interactions (dashed bonds) for I. Symmetry code (iii) -x + 1, -y + 1, -z + 1. Non-relavent hydrogen atoms and phenyl rings have been omitted for clarity.

**Fig. 3.**
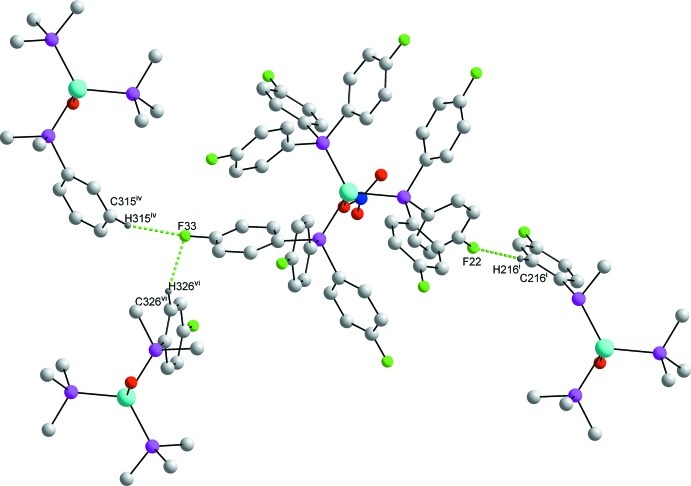
Intermolecular C—H···F (dashed bonds) for I. Symmetry codes (i) x - 1, y + 1, z, (iv) x, y - 1, z and (vi) -x + 2, -y + 2, -z. Non-relevant hydrogen atoms and phenyl rings have been omitted for clarity.

**Fig. 4.**
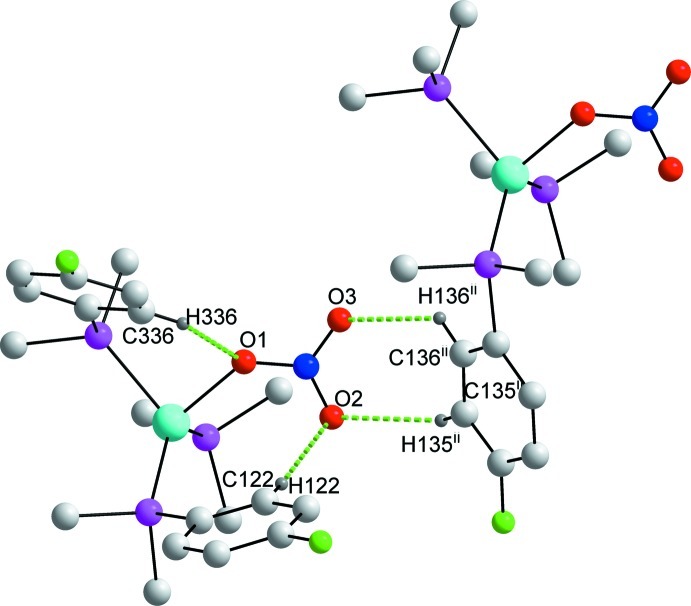
Inter- and intramolecular C—H···O interactions (dashed bonds) for I. Symmetry code (iii) x - 1, y, z. Non-relevant hydrogen atoms and phenyl rings have been omitted for clarity.

## supplementary crystallographic information

### Comment

The title compound (I) has a copper(I) metal center co-ordinated by three tris-4-fluorophenylphosphane ligands ((*p*-FPh)_3_P) and a nitrato ligand. The ligating atoms define a distorted trigonal pyramid which is similar to what was found for [Cu(PPh_3_)_3_(X)] X = ClO_4_^-^, BF_4_^-^,NO_3_^-^, HCO_2_^-^ (Hanna *et al.*, 2005) complexes, where the average P—Cu—P angles are in the range 112.29 (4)° - 121.37 (6)°. While markedly different, the P—Cu—P bond angles for for I (Table 1) fall within this range with an average of 116 (2)°. The dissimilarity observed for the O—Cu—P bond angles (Table 1) are as a result of C—H···O (Figure 4) and O2···π (centroid C221-C226) interactions contributing to the non-linearity of the N1—O1—Cu1 angle and the deviation (14.39 (5)°) of the nitrato ligand from the axial position. The average Cu—P bond lengths for [Cu(PPh_3_)_3_(NO_3_)].EtOH (Dyason *et al.*, 1986), [Cu(PPh_3_)_3_(NO_3_)].MeOH (Steyl, 2009) and I were observed to be 2.329 (9) Å, 2.326 (10) Å and 2.300 (13) Å respectively.

An intermolecular π–π interaction is observed for A (centroid C131—C136)···B (centroid C211—C216) with a distance of 3.6113 (11) Å and is stabilized by the bifurcated hydrogen fluorine interaction C215—H215···F21^iii^ and C215—H215···F13^iii^ with H···F distances of 2.61 Å and 2.51 Å respectively (see Figure 2). An additional stabilizing effect arises from the C135^ii^—H135^ii^···O2 and C136^ii^—H136^ii^···O3 interactions (Figure 4) with H···O distances of 2.58 Å and 2.53 Å. Additional C—H···F interactions are illustrated in Figure 3. All of these interactions contribute to tight packing of I.

### Experimental

tris-4-fluorophenylphosphane (2 mmol) was added to a solution of CuNO_3_ (1 mmol) in warm MeOH (15 ml, 70 °C) and the resulting solution was stirred for *c.a.* 1 h. The solution was filtered and allowed to cool slowly. Crystals suitable for single-crystal X-ray diffraction were obtained from the slow evaporation of the solution.

### Refinement

All H atoms were placed in geometrically idealized positions and constrained to ride on their parent atoms, with C—H = 0.95 Å and *U*_iso_(H) = 1.2 *U*_eq_(C) for aromatic H atoms.

### Crystal data

**Table:**

[Cu(NO_3_)(C_18_H_12_F_3_P)_3_]	*Z* = 2
*M**_r_* = 1074.29	*F*(000) = 1092
Triclinic, *P*1	*D*_x_ = 1.501 Mg m^−^^3^
Hall symbol: -P 1	Mo *K*α radiation, λ = 0.71073 Å
*a* = 9.3861 (3) Å	Cell parameters from 9076 reflections
*b* = 12.2552 (4) Å	θ = 2.5–28.2°
*c* = 21.4820 (7) Å	µ = 0.64 mm^−^^1^
α = 85.274 (2)°	*T* = 100 K
β = 86.843 (1)°	Column, colourless
γ = 74.954 (1)°	0.38 × 0.11 × 0.08 mm
*V* = 2376.76 (13) Å^3^	

### Data collection

**Table:**

Bruker X8 APEXII 4K KappaCCD diffractometer	11760 independent reflections
Radiation source: sealed tube	9439 reflections with *I* > 2σ(*I*)
Graphite monochromator	*R*_int_ = 0.030
Detector resolution: 512 pixels mm^-1^	θ_max_ = 28.5°, θ_min_ = 2.4°
φ and ω scans	*h* = −9→12
Absorption correction: multi-scan (*SADABS*; Bruker, 2004)	*k* = −16→16
*T*_min_ = 0.792, *T*_max_ = 0.950	*l* = −28→27
28458 measured reflections	

### Refinement

**Table:**

Refinement on *F*^2^	0 restraints
Least-squares matrix: full	H-atom parameters constrained
*R*[*F*^2^ > 2σ(*F*^2^)] = 0.035	*w* = 1/[σ^2^(*F*_o_^2^) + (0.0333*P*)^2^ + 0.8637*P*] where *P* = (*F*_o_^2^ + 2*F*_c_^2^)/3
*wR*(*F*^2^) = 0.085	(Δ/σ)_max_ = 0.001
*S* = 1.05	Δρ_max_ = 0.40 e Å^−^^3^
11760 reflections	Δρ_min_ = −0.41 e Å^−^^3^
640 parameters	

### Special details

**Table:**

Geometry. All e.s.d.'s (except the e.s.d. in the dihedral angle between two l.s. planes) are estimated using the full covariance matrix. The cell e.s.d.'s are taken into account individually in the estimation of e.s.d.'s in distances, angles and torsion angles; correlations between e.s.d.'s in cell parameters are only used when they are defined by crystal symmetry. An approximate (isotropic) treatment of cell e.s.d.'s is used for estimating e.s.d.'s involving l.s. planes.

### Fractional atomic coordinates and isotropic or equivalent isotropic displacement parameters (Å^2^)

**Table:**

	*x*	*y*	*z*	*U*_iso_*/*U*_eq_	
C111	0.64094 (18)	1.01573 (14)	0.27195 (8)	0.0156 (3)	
C112	0.68685 (19)	1.07502 (15)	0.21964 (9)	0.0188 (4)	
H112	0.7894	1.0632	0.2097	0.023*	
C113	0.5860 (2)	1.15064 (16)	0.18194 (9)	0.0214 (4)	
H113	0.6179	1.1905	0.1463	0.026*	
C114	0.4384 (2)	1.16645 (16)	0.19755 (9)	0.0224 (4)	
C115	0.3879 (2)	1.11255 (17)	0.24917 (9)	0.0236 (4)	
H115	0.2851	1.1269	0.2592	0.028*	
C116	0.49013 (19)	1.03644 (16)	0.28659 (9)	0.0200 (4)	
H116	0.4569	0.9981	0.3225	0.024*	
C121	0.88511 (18)	0.99336 (15)	0.35054 (8)	0.0164 (4)	
C122	1.0241 (2)	0.93895 (17)	0.37273 (9)	0.0235 (4)	
H122	1.0636	0.8604	0.3679	0.028*	
C123	1.1054 (2)	0.99874 (18)	0.40190 (10)	0.0302 (5)	
H123	1.2002	0.9621	0.4172	0.036*	
C124	1.0452 (2)	1.11196 (18)	0.40800 (10)	0.0300 (5)	
C125	0.9084 (2)	1.16862 (18)	0.38719 (11)	0.0338 (5)	
H125	0.8696	1.2471	0.3925	0.041*	
C126	0.8282 (2)	1.10793 (16)	0.35806 (10)	0.0261 (4)	
H126	0.7333	1.1454	0.3431	0.031*	
C131	0.67292 (18)	0.86418 (15)	0.38106 (8)	0.0155 (3)	
C132	0.67507 (19)	0.89769 (15)	0.44123 (8)	0.0183 (4)	
H132	0.7372	0.9443	0.4497	0.022*	
C133	0.5868 (2)	0.86338 (16)	0.48895 (9)	0.0217 (4)	
H133	0.5892	0.885	0.5303	0.026*	
C134	0.4963 (2)	0.79770 (16)	0.47510 (9)	0.0214 (4)	
C135	0.49013 (19)	0.76214 (15)	0.41629 (9)	0.0202 (4)	
H135	0.4258	0.717	0.4082	0.024*	
C136	0.58129 (19)	0.79473 (15)	0.36951 (9)	0.0177 (4)	
H136	0.5817	0.7695	0.3288	0.021*	
C211	0.84201 (19)	0.55517 (15)	0.36385 (8)	0.0169 (4)	
C212	0.88652 (19)	0.60020 (16)	0.41492 (9)	0.0203 (4)	
H212	0.956	0.6446	0.4086	0.024*	
C213	0.8305 (2)	0.58076 (18)	0.47462 (9)	0.0259 (4)	
H213	0.861	0.6106	0.5095	0.031*	
C214	0.7292 (2)	0.51683 (18)	0.48172 (9)	0.0286 (5)	
C215	0.6808 (2)	0.47230 (17)	0.43309 (10)	0.0296 (5)	
H215	0.6102	0.429	0.4399	0.036*	
C216	0.7382 (2)	0.49225 (16)	0.37315 (9)	0.0225 (4)	
H216	0.706	0.4626	0.3385	0.027*	
C221	1.10916 (18)	0.48680 (15)	0.29250 (8)	0.0161 (4)	
C222	1.2149 (2)	0.49938 (16)	0.24626 (9)	0.0225 (4)	
H222	1.1869	0.5508	0.2108	0.027*	
C223	1.3602 (2)	0.43753 (17)	0.25149 (11)	0.0288 (5)	
H223	1.4323	0.4455	0.2198	0.035*	
C224	1.3979 (2)	0.36487 (17)	0.30303 (10)	0.0269 (4)	
C225	1.2974 (2)	0.34649 (18)	0.34868 (10)	0.0297 (5)	
H225	1.3265	0.2931	0.3832	0.036*	
C226	1.1516 (2)	0.40819 (17)	0.34301 (9)	0.0249 (4)	
H226	1.0797	0.3967	0.374	0.03*	
C231	0.83035 (18)	0.52013 (15)	0.23431 (8)	0.0155 (3)	
C232	0.70057 (19)	0.58837 (16)	0.20949 (8)	0.0182 (4)	
H232	0.6622	0.6624	0.2232	0.022*	
C233	0.6262 (2)	0.55066 (17)	0.16533 (9)	0.0236 (4)	
H233	0.5381	0.5978	0.1482	0.028*	
C234	0.6842 (2)	0.44263 (18)	0.14708 (9)	0.0238 (4)	
C235	0.8096 (2)	0.37125 (17)	0.17122 (10)	0.0263 (4)	
H235	0.8451	0.2964	0.1582	0.032*	
C236	0.8839 (2)	0.41083 (16)	0.21519 (9)	0.0224 (4)	
H236	0.9716	0.363	0.2322	0.027*	
C311	1.04880 (18)	0.69465 (14)	0.10744 (8)	0.0140 (3)	
C312	1.18958 (18)	0.70551 (15)	0.08908 (8)	0.0168 (4)	
H312	1.2172	0.7721	0.0972	0.02*	
C313	1.2899 (2)	0.62024 (16)	0.05907 (9)	0.0214 (4)	
H313	1.3852	0.6283	0.0459	0.026*	
C314	1.2481 (2)	0.52395 (16)	0.04881 (9)	0.0212 (4)	
C315	1.1117 (2)	0.50835 (16)	0.06709 (9)	0.0224 (4)	
H315	1.0865	0.4404	0.0598	0.027*	
C316	1.0117 (2)	0.59482 (15)	0.09661 (9)	0.0192 (4)	
H316	0.9168	0.5858	0.1096	0.023*	
C321	0.74569 (18)	0.81865 (14)	0.10980 (8)	0.0144 (3)	
C322	0.61174 (19)	0.85818 (15)	0.14206 (9)	0.0177 (4)	
H322	0.6108	0.8702	0.1852	0.021*	
C323	0.4793 (2)	0.88015 (16)	0.11152 (10)	0.0244 (4)	
H323	0.3876	0.9072	0.1332	0.029*	
C324	0.4848 (2)	0.86173 (16)	0.04930 (10)	0.0241 (4)	
C325	0.6133 (2)	0.82046 (17)	0.01569 (9)	0.0248 (4)	
H325	0.6123	0.8071	−0.0272	0.03*	
C326	0.7452 (2)	0.79875 (16)	0.04667 (8)	0.0196 (4)	
H326	0.8361	0.7701	0.0246	0.024*	
C331	0.96604 (18)	0.93534 (14)	0.12332 (8)	0.0137 (3)	
C332	0.8948 (2)	1.00834 (15)	0.07499 (9)	0.0200 (4)	
H332	0.8237	0.9868	0.0522	0.024*	
C333	0.9262 (2)	1.11221 (16)	0.05949 (9)	0.0225 (4)	
H333	0.8762	1.1625	0.027	0.027*	
C334	1.0311 (2)	1.13997 (15)	0.09233 (9)	0.0190 (4)	
C335	1.1049 (2)	1.07060 (16)	0.14004 (9)	0.0210 (4)	
H335	1.1774	1.0924	0.1618	0.025*	
C336	1.07114 (19)	0.96782 (15)	0.15582 (9)	0.0182 (4)	
H336	1.1201	0.9191	0.1891	0.022*	
N1	1.24915 (16)	0.72517 (14)	0.29043 (8)	0.0232 (4)	
O1	1.14700 (13)	0.76757 (11)	0.25203 (6)	0.0228 (3)	
O2	1.21663 (16)	0.68829 (12)	0.34380 (7)	0.0288 (3)	
O3	1.37844 (15)	0.71966 (15)	0.27340 (8)	0.0413 (4)	
F11	0.33854 (12)	1.23879 (10)	0.16002 (6)	0.0319 (3)	
F12	1.12332 (15)	1.17149 (11)	0.43652 (7)	0.0458 (4)	
F13	0.40845 (13)	0.76620 (10)	0.52191 (6)	0.0330 (3)	
F21	0.67367 (15)	0.49784 (12)	0.54008 (6)	0.0447 (4)	
F22	1.54253 (12)	0.30798 (11)	0.30944 (7)	0.0418 (3)	
F23	0.61304 (14)	0.40369 (11)	0.10379 (6)	0.0355 (3)	
F31	1.34503 (13)	0.44073 (10)	0.01912 (6)	0.0320 (3)	
F32	0.35528 (12)	0.88451 (11)	0.01908 (6)	0.0364 (3)	
F33	1.06177 (12)	1.24204 (9)	0.07802 (5)	0.0261 (3)	
P1	0.78177 (5)	0.90846 (4)	0.31532 (2)	0.01394 (9)	
P2	0.92541 (5)	0.58263 (4)	0.28780 (2)	0.01375 (9)	
P3	0.91661 (5)	0.80309 (4)	0.15007 (2)	0.01231 (9)	
Cu1	0.92130 (2)	0.766649 (17)	0.258012 (10)	0.01294 (6)	

### Atomic displacement parameters (Å^2^)

**Table:**

	*U*^11^	*U*^22^	*U*^33^	*U*^12^	*U*^13^	*U*^23^
C111	0.0158 (8)	0.0129 (9)	0.0179 (9)	−0.0022 (7)	−0.0035 (7)	−0.0036 (7)
C112	0.0167 (8)	0.0160 (9)	0.0234 (10)	−0.0028 (7)	−0.0020 (7)	−0.0035 (7)
C113	0.0265 (10)	0.0166 (9)	0.0217 (10)	−0.0066 (8)	−0.0040 (8)	0.0000 (7)
C114	0.0231 (9)	0.0154 (9)	0.0281 (11)	−0.0017 (8)	−0.0144 (8)	0.0006 (8)
C115	0.0145 (8)	0.0253 (11)	0.0304 (11)	−0.0027 (8)	−0.0071 (8)	−0.0021 (8)
C116	0.0172 (8)	0.0226 (10)	0.0202 (10)	−0.0046 (8)	−0.0029 (7)	−0.0012 (7)
C121	0.0139 (8)	0.0164 (9)	0.0190 (9)	−0.0034 (7)	−0.0020 (7)	−0.0020 (7)
C122	0.0201 (9)	0.0185 (10)	0.0297 (11)	0.0007 (8)	−0.0072 (8)	−0.0029 (8)
C123	0.0207 (9)	0.0300 (12)	0.0405 (13)	−0.0047 (9)	−0.0131 (9)	−0.0029 (9)
C124	0.0306 (11)	0.0278 (11)	0.0375 (12)	−0.0154 (9)	−0.0142 (9)	−0.0013 (9)
C125	0.0368 (12)	0.0165 (10)	0.0497 (14)	−0.0053 (9)	−0.0207 (10)	−0.0040 (9)
C126	0.0228 (9)	0.0181 (10)	0.0370 (12)	−0.0013 (8)	−0.0143 (9)	−0.0036 (8)
C131	0.0134 (8)	0.0139 (9)	0.0173 (9)	−0.0002 (7)	−0.0016 (7)	0.0001 (7)
C132	0.0196 (9)	0.0149 (9)	0.0199 (9)	−0.0026 (7)	−0.0021 (7)	−0.0027 (7)
C133	0.0258 (10)	0.0186 (10)	0.0177 (10)	0.0002 (8)	−0.0014 (7)	−0.0020 (7)
C134	0.0200 (9)	0.0182 (10)	0.0231 (10)	−0.0021 (8)	0.0053 (7)	0.0026 (7)
C135	0.0162 (8)	0.0158 (9)	0.0276 (10)	−0.0029 (7)	−0.0026 (7)	0.0012 (7)
C136	0.0167 (8)	0.0150 (9)	0.0204 (9)	−0.0012 (7)	−0.0023 (7)	−0.0025 (7)
C211	0.0161 (8)	0.0134 (9)	0.0179 (9)	0.0010 (7)	0.0020 (7)	0.0012 (7)
C212	0.0165 (8)	0.0199 (10)	0.0211 (10)	0.0007 (7)	−0.0007 (7)	0.0019 (7)
C213	0.0228 (9)	0.0292 (11)	0.0185 (10)	0.0056 (8)	−0.0010 (8)	0.0001 (8)
C214	0.0302 (11)	0.0268 (11)	0.0197 (10)	0.0042 (9)	0.0103 (8)	0.0067 (8)
C215	0.0323 (11)	0.0214 (11)	0.0338 (12)	−0.0083 (9)	0.0142 (9)	0.0013 (9)
C216	0.0258 (10)	0.0150 (9)	0.0253 (10)	−0.0041 (8)	0.0049 (8)	−0.0005 (8)
C221	0.0153 (8)	0.0129 (9)	0.0197 (9)	−0.0024 (7)	−0.0007 (7)	−0.0022 (7)
C222	0.0188 (9)	0.0181 (10)	0.0278 (11)	−0.0018 (8)	0.0018 (8)	0.0034 (8)
C223	0.0175 (9)	0.0226 (11)	0.0441 (13)	−0.0038 (8)	0.0080 (9)	−0.0001 (9)
C224	0.0145 (9)	0.0202 (10)	0.0429 (13)	0.0040 (8)	−0.0076 (8)	−0.0083 (9)
C225	0.0301 (11)	0.0255 (11)	0.0249 (11)	0.0083 (9)	−0.0057 (9)	0.0002 (8)
C226	0.0248 (10)	0.0229 (11)	0.0212 (10)	0.0030 (8)	0.0021 (8)	0.0011 (8)
C231	0.0146 (8)	0.0160 (9)	0.0162 (9)	−0.0052 (7)	0.0026 (7)	−0.0002 (7)
C232	0.0187 (8)	0.0163 (9)	0.0204 (9)	−0.0068 (7)	0.0009 (7)	0.0014 (7)
C233	0.0231 (9)	0.0255 (11)	0.0241 (10)	−0.0110 (8)	−0.0043 (8)	0.0044 (8)
C234	0.0273 (10)	0.0337 (12)	0.0169 (10)	−0.0189 (9)	−0.0003 (8)	−0.0031 (8)
C235	0.0235 (9)	0.0238 (11)	0.0344 (12)	−0.0089 (8)	0.0054 (8)	−0.0143 (9)
C236	0.0167 (9)	0.0191 (10)	0.0305 (11)	−0.0021 (7)	0.0020 (8)	−0.0073 (8)
C311	0.0144 (8)	0.0148 (9)	0.0121 (8)	−0.0030 (7)	−0.0020 (6)	0.0013 (6)
C312	0.0154 (8)	0.0153 (9)	0.0196 (9)	−0.0040 (7)	−0.0021 (7)	0.0003 (7)
C313	0.0146 (8)	0.0218 (10)	0.0266 (10)	−0.0028 (7)	0.0005 (7)	−0.0012 (8)
C314	0.0225 (9)	0.0176 (10)	0.0202 (10)	0.0013 (8)	0.0009 (7)	−0.0039 (7)
C315	0.0290 (10)	0.0154 (9)	0.0242 (10)	−0.0087 (8)	0.0022 (8)	−0.0028 (7)
C316	0.0199 (9)	0.0174 (9)	0.0214 (10)	−0.0077 (7)	0.0023 (7)	−0.0005 (7)
C321	0.0147 (8)	0.0135 (8)	0.0164 (9)	−0.0067 (7)	−0.0028 (6)	0.0019 (7)
C322	0.0167 (8)	0.0159 (9)	0.0213 (10)	−0.0052 (7)	−0.0024 (7)	−0.0015 (7)
C323	0.0131 (8)	0.0223 (10)	0.0379 (12)	−0.0049 (8)	−0.0032 (8)	−0.0008 (8)
C324	0.0190 (9)	0.0205 (10)	0.0358 (12)	−0.0108 (8)	−0.0165 (8)	0.0094 (8)
C325	0.0312 (10)	0.0270 (11)	0.0207 (10)	−0.0155 (9)	−0.0107 (8)	0.0048 (8)
C326	0.0200 (9)	0.0232 (10)	0.0177 (9)	−0.0098 (8)	−0.0030 (7)	0.0020 (7)
C331	0.0133 (8)	0.0128 (8)	0.0152 (9)	−0.0039 (7)	0.0030 (6)	−0.0024 (6)
C332	0.0233 (9)	0.0187 (10)	0.0199 (10)	−0.0087 (8)	−0.0051 (7)	0.0013 (7)
C333	0.0300 (10)	0.0178 (10)	0.0191 (10)	−0.0063 (8)	−0.0041 (8)	0.0051 (7)
C334	0.0224 (9)	0.0125 (9)	0.0225 (10)	−0.0066 (7)	0.0099 (7)	−0.0033 (7)
C335	0.0178 (9)	0.0188 (10)	0.0290 (11)	−0.0086 (8)	−0.0003 (7)	−0.0044 (8)
C336	0.0155 (8)	0.0170 (9)	0.0226 (10)	−0.0049 (7)	−0.0034 (7)	0.0003 (7)
N1	0.0156 (7)	0.0231 (9)	0.0315 (10)	−0.0032 (7)	−0.0046 (7)	−0.0089 (7)
O1	0.0144 (6)	0.0267 (7)	0.0269 (7)	−0.0059 (6)	−0.0067 (5)	0.0062 (6)
O2	0.0338 (8)	0.0245 (8)	0.0250 (8)	−0.0007 (6)	−0.0099 (6)	0.0000 (6)
O3	0.0131 (7)	0.0582 (11)	0.0545 (11)	−0.0075 (7)	−0.0034 (7)	−0.0180 (9)
F11	0.0277 (6)	0.0272 (7)	0.0392 (7)	−0.0039 (5)	−0.0192 (5)	0.0091 (5)
F12	0.0462 (8)	0.0346 (8)	0.0653 (10)	−0.0196 (6)	−0.0318 (7)	−0.0038 (7)
F13	0.0361 (7)	0.0334 (7)	0.0298 (7)	−0.0134 (6)	0.0123 (5)	0.0017 (5)
F21	0.0517 (8)	0.0537 (9)	0.0229 (7)	−0.0095 (7)	0.0156 (6)	0.0076 (6)
F22	0.0165 (6)	0.0341 (8)	0.0685 (10)	0.0086 (5)	−0.0121 (6)	−0.0083 (7)
F23	0.0444 (7)	0.0440 (8)	0.0280 (7)	−0.0263 (6)	−0.0070 (6)	−0.0078 (6)
F31	0.0287 (6)	0.0238 (6)	0.0409 (7)	−0.0006 (5)	0.0087 (5)	−0.0140 (5)
F32	0.0242 (6)	0.0418 (8)	0.0470 (8)	−0.0159 (6)	−0.0236 (5)	0.0143 (6)
F33	0.0366 (6)	0.0156 (6)	0.0289 (6)	−0.0135 (5)	0.0089 (5)	−0.0020 (5)
P1	0.0130 (2)	0.0125 (2)	0.0160 (2)	−0.00207 (17)	−0.00181 (16)	−0.00245 (17)
P2	0.0129 (2)	0.0121 (2)	0.0153 (2)	−0.00204 (17)	−0.00006 (16)	0.00020 (17)
P3	0.01112 (19)	0.0127 (2)	0.0135 (2)	−0.00410 (17)	−0.00106 (16)	0.00032 (16)
Cu1	0.01211 (10)	0.01249 (11)	0.01381 (11)	−0.00253 (8)	−0.00091 (8)	−0.00025 (8)

### Geometric parameters (Å, º)

**Table:**

C111—C116	1.394 (2)	C225—H225	0.95
C111—C112	1.397 (3)	C226—H226	0.95
C111—P1	1.8372 (18)	C231—C236	1.391 (2)
C112—C113	1.384 (3)	C231—C232	1.392 (2)
C112—H112	0.95	C231—P2	1.8209 (18)
C113—C114	1.375 (3)	C232—C233	1.383 (3)
C113—H113	0.95	C232—H232	0.95
C114—F11	1.359 (2)	C233—C234	1.375 (3)
C114—C115	1.369 (3)	C233—H233	0.95
C115—C116	1.391 (3)	C234—F23	1.359 (2)
C115—H115	0.95	C234—C235	1.370 (3)
C116—H116	0.95	C235—C236	1.392 (3)
C121—C126	1.386 (3)	C235—H235	0.95
C121—C122	1.392 (2)	C236—H236	0.95
C121—P1	1.8249 (17)	C311—C312	1.395 (2)
C122—C123	1.389 (3)	C311—C316	1.396 (2)
C122—H122	0.95	C311—P3	1.8340 (18)
C123—C124	1.370 (3)	C312—C313	1.388 (3)
C123—H123	0.95	C312—H312	0.95
C124—F12	1.359 (2)	C313—C314	1.374 (3)
C124—C125	1.371 (3)	C313—H313	0.95
C125—C126	1.390 (3)	C314—F31	1.355 (2)
C125—H125	0.95	C314—C315	1.375 (3)
C126—H126	0.95	C315—C316	1.391 (3)
C131—C132	1.391 (2)	C315—H315	0.95
C131—C136	1.401 (2)	C316—H316	0.95
C131—P1	1.8263 (18)	C321—C322	1.392 (2)
C132—C133	1.389 (3)	C321—C326	1.398 (2)
C132—H132	0.95	C321—P3	1.8236 (17)
C133—C134	1.371 (3)	C322—C323	1.391 (2)
C133—H133	0.95	C322—H322	0.95
C134—F13	1.360 (2)	C323—C324	1.370 (3)
C134—C135	1.379 (3)	C323—H323	0.95
C135—C136	1.385 (2)	C324—F32	1.363 (2)
C135—H135	0.95	C324—C325	1.371 (3)
C136—H136	0.95	C325—C326	1.392 (2)
C211—C216	1.389 (2)	C325—H325	0.95
C211—C212	1.397 (3)	C326—H326	0.95
C211—P2	1.8166 (18)	C331—C332	1.392 (2)
C212—C213	1.385 (3)	C331—C336	1.394 (2)
C212—H212	0.95	C331—P3	1.8385 (17)
C213—C214	1.376 (3)	C332—C333	1.390 (2)
C213—H213	0.95	C332—H332	0.95
C214—F21	1.357 (2)	C333—C334	1.368 (3)
C214—C215	1.367 (3)	C333—H333	0.95
C215—C216	1.396 (3)	C334—F33	1.3624 (19)
C215—H215	0.95	C334—C335	1.372 (3)
C216—H216	0.95	C335—C336	1.388 (2)
C221—C226	1.393 (3)	C335—H335	0.95
C221—C222	1.393 (2)	C336—H336	0.95
C221—P2	1.8199 (18)	N1—O3	1.234 (2)
C222—C223	1.383 (3)	N1—O2	1.249 (2)
C222—H222	0.95	N1—O1	1.2757 (19)
C223—C224	1.364 (3)	O1—Cu1	2.1182 (12)
C223—H223	0.95	P1—Cu1	2.2901 (5)
C224—F22	1.364 (2)	P2—Cu1	2.2840 (5)
C224—C225	1.370 (3)	P3—Cu1	2.3256 (5)
C225—C226	1.387 (3)		
			
C116—C111—C112	118.43 (16)	C231—C232—H232	119.3
C116—C111—P1	123.28 (14)	C234—C233—C232	117.67 (18)
C112—C111—P1	118.20 (13)	C234—C233—H233	121.2
C113—C112—C111	121.34 (17)	C232—C233—H233	121.2
C113—C112—H112	119.3	F23—C234—C235	118.14 (18)
C111—C112—H112	119.3	F23—C234—C233	118.74 (18)
C114—C113—C112	118.08 (18)	C235—C234—C233	123.11 (17)
C114—C113—H113	121	C234—C235—C236	118.50 (18)
C112—C113—H113	121	C234—C235—H235	120.8
F11—C114—C115	118.73 (17)	C236—C235—H235	120.8
F11—C114—C113	118.43 (17)	C231—C236—C235	120.30 (18)
C115—C114—C113	122.83 (17)	C231—C236—H236	119.8
C114—C115—C116	118.59 (17)	C235—C236—H236	119.8
C114—C115—H115	120.7	C312—C311—C316	118.70 (16)
C116—C115—H115	120.7	C312—C311—P3	121.87 (13)
C115—C116—C111	120.70 (18)	C316—C311—P3	119.29 (13)
C115—C116—H116	119.7	C313—C312—C311	120.86 (16)
C111—C116—H116	119.7	C313—C312—H312	119.6
C126—C121—C122	119.30 (16)	C311—C312—H312	119.6
C126—C121—P1	122.57 (13)	C314—C313—C312	118.43 (16)
C122—C121—P1	118.08 (14)	C314—C313—H313	120.8
C123—C122—C121	120.47 (18)	C312—C313—H313	120.8
C123—C122—H122	119.8	F31—C314—C313	118.77 (16)
C121—C122—H122	119.8	F31—C314—C315	118.34 (17)
C124—C123—C122	118.28 (18)	C313—C314—C315	122.88 (17)
C124—C123—H123	120.9	C314—C315—C316	118.14 (17)
C122—C123—H123	120.9	C314—C315—H315	120.9
F12—C124—C123	118.94 (18)	C316—C315—H315	120.9
F12—C124—C125	117.93 (19)	C315—C316—C311	120.98 (16)
C123—C124—C125	123.13 (18)	C315—C316—H316	119.5
C124—C125—C126	118.07 (19)	C311—C316—H316	119.5
C124—C125—H125	121	C322—C321—C326	119.14 (16)
C126—C125—H125	121	C322—C321—P3	118.73 (13)
C121—C126—C125	120.75 (18)	C326—C321—P3	122.01 (13)
C121—C126—H126	119.6	C323—C322—C321	120.44 (17)
C125—C126—H126	119.6	C323—C322—H322	119.8
C132—C131—C136	118.98 (16)	C321—C322—H322	119.8
C132—C131—P1	123.15 (13)	C324—C323—C322	118.23 (17)
C136—C131—P1	117.86 (13)	C324—C323—H323	120.9
C133—C132—C131	120.39 (16)	C322—C323—H323	120.9
C133—C132—H132	119.8	F32—C324—C323	118.35 (18)
C131—C132—H132	119.8	F32—C324—C325	117.95 (18)
C134—C133—C132	118.55 (17)	C323—C324—C325	123.70 (17)
C134—C133—H133	120.7	C324—C325—C326	117.62 (18)
C132—C133—H133	120.7	C324—C325—H325	121.2
F13—C134—C133	118.11 (17)	C326—C325—H325	121.2
F13—C134—C135	118.56 (16)	C325—C326—C321	120.84 (17)
C133—C134—C135	123.33 (17)	C325—C326—H326	119.6
C134—C135—C136	117.47 (16)	C321—C326—H326	119.6
C134—C135—H135	121.3	C332—C331—C336	118.71 (16)
C136—C135—H135	121.3	C332—C331—P3	122.55 (13)
C135—C136—C131	121.24 (17)	C336—C331—P3	118.56 (13)
C135—C136—H136	119.4	C333—C332—C331	121.02 (16)
C131—C136—H136	119.4	C333—C332—H332	119.5
C216—C211—C212	119.37 (17)	C331—C332—H332	119.5
C216—C211—P2	123.31 (14)	C334—C333—C332	118.15 (17)
C212—C211—P2	117.31 (13)	C334—C333—H333	120.9
C213—C212—C211	120.73 (18)	C332—C333—H333	120.9
C213—C212—H212	119.6	F33—C334—C333	118.72 (17)
C211—C212—H212	119.6	F33—C334—C335	118.29 (16)
C214—C213—C212	117.87 (19)	C333—C334—C335	122.97 (17)
C214—C213—H213	121.1	C334—C335—C336	118.45 (17)
C212—C213—H213	121.1	C334—C335—H335	120.8
F21—C214—C215	118.41 (19)	C336—C335—H335	120.8
F21—C214—C213	118.1 (2)	C335—C336—C331	120.68 (17)
C215—C214—C213	123.51 (18)	C335—C336—H336	119.7
C214—C215—C216	118.15 (19)	C331—C336—H336	119.7
C214—C215—H215	120.9	O3—N1—O2	121.54 (17)
C216—C215—H215	120.9	O3—N1—O1	118.99 (17)
C211—C216—C215	120.35 (19)	O2—N1—O1	119.45 (15)
C211—C216—H216	119.8	N1—O1—Cu1	129.87 (11)
C215—C216—H216	119.8	C121—P1—C131	103.52 (8)
C226—C221—C222	118.76 (17)	C121—P1—C111	102.89 (8)
C226—C221—P2	122.63 (14)	C131—P1—C111	101.99 (8)
C222—C221—P2	118.38 (14)	C121—P1—Cu1	115.18 (6)
C223—C222—C221	120.54 (18)	C131—P1—Cu1	116.00 (6)
C223—C222—H222	119.7	C111—P1—Cu1	115.38 (6)
C221—C222—H222	119.7	C211—P2—C221	102.44 (8)
C224—C223—C222	118.67 (18)	C211—P2—C231	103.80 (8)
C224—C223—H223	120.7	C221—P2—C231	104.60 (8)
C222—C223—H223	120.7	C211—P2—Cu1	117.00 (6)
F22—C224—C223	118.50 (18)	C221—P2—Cu1	114.73 (6)
F22—C224—C225	118.46 (19)	C231—P2—Cu1	112.79 (6)
C223—C224—C225	123.04 (18)	C321—P3—C311	102.69 (8)
C224—C225—C226	118.02 (19)	C321—P3—C331	101.81 (8)
C224—C225—H225	121	C311—P3—C331	103.93 (8)
C226—C225—H225	121	C321—P3—Cu1	119.88 (6)
C225—C226—C221	120.88 (18)	C311—P3—Cu1	113.47 (6)
C225—C226—H226	119.6	C331—P3—Cu1	113.18 (6)
C221—C226—H226	119.6	O1—Cu1—P2	103.96 (4)
C236—C231—C232	118.97 (16)	O1—Cu1—P1	112.11 (4)
C236—C231—P2	123.58 (14)	P2—Cu1—P1	119.537 (18)
C232—C231—P2	117.38 (13)	O1—Cu1—P3	87.93 (4)
C233—C232—C231	121.42 (18)	P2—Cu1—P3	112.289 (18)
C233—C232—H232	119.3	P1—Cu1—P3	115.765 (18)
			
C116—C111—C112—C113	−1.6 (3)	C332—C331—C336—C335	−0.4 (3)
P1—C111—C112—C113	174.93 (14)	P3—C331—C336—C335	−175.65 (14)
C111—C112—C113—C114	0.3 (3)	O3—N1—O1—Cu1	168.52 (13)
C112—C113—C114—F11	−178.64 (16)	O2—N1—O1—Cu1	−10.1 (2)
C112—C113—C114—C115	1.4 (3)	C126—C121—P1—C131	−86.16 (17)
F11—C114—C115—C116	178.39 (16)	C122—C121—P1—C131	91.29 (16)
C113—C114—C115—C116	−1.6 (3)	C126—C121—P1—C111	19.76 (18)
C114—C115—C116—C111	0.2 (3)	C122—C121—P1—C111	−162.80 (15)
C112—C111—C116—C115	1.4 (3)	C126—C121—P1—Cu1	146.16 (15)
P1—C111—C116—C115	−175.00 (14)	C122—C121—P1—Cu1	−36.39 (17)
C126—C121—C122—C123	−0.2 (3)	C132—C131—P1—C121	2.17 (17)
P1—C121—C122—C123	−177.78 (16)	C136—C131—P1—C121	−178.98 (14)
C121—C122—C123—C124	−0.1 (3)	C132—C131—P1—C111	−104.43 (15)
C122—C123—C124—F12	179.89 (19)	C136—C131—P1—C111	74.42 (15)
C122—C123—C124—C125	0.6 (4)	C132—C131—P1—Cu1	129.34 (14)
F12—C124—C125—C126	−179.9 (2)	C136—C131—P1—Cu1	−51.81 (15)
C123—C124—C125—C126	−0.6 (4)	C116—C111—P1—C121	−115.02 (15)
C122—C121—C126—C125	0.2 (3)	C112—C111—P1—C121	68.61 (15)
P1—C121—C126—C125	177.63 (17)	C116—C111—P1—C131	−7.93 (17)
C124—C125—C126—C121	0.2 (3)	C112—C111—P1—C131	175.70 (14)
C136—C131—C132—C133	−0.5 (3)	C116—C111—P1—Cu1	118.70 (14)
P1—C131—C132—C133	178.38 (14)	C112—C111—P1—Cu1	−57.67 (15)
C131—C132—C133—C134	−1.1 (3)	C216—C211—P2—C221	−104.51 (16)
C132—C133—C134—F13	−178.86 (16)	C212—C211—P2—C221	75.32 (15)
C132—C133—C134—C135	1.2 (3)	C216—C211—P2—C231	4.16 (18)
F13—C134—C135—C136	−179.62 (16)	C212—C211—P2—C231	−176.01 (14)
C133—C134—C135—C136	0.4 (3)	C216—C211—P2—Cu1	129.11 (14)
C134—C135—C136—C131	−2.0 (3)	C212—C211—P2—Cu1	−51.06 (15)
C132—C131—C136—C135	2.0 (3)	C226—C221—P2—C211	6.02 (17)
P1—C131—C136—C135	−176.86 (14)	C222—C221—P2—C211	−168.41 (14)
C216—C211—C212—C213	1.3 (3)	C226—C221—P2—C231	−102.03 (16)
P2—C211—C212—C213	−178.51 (14)	C222—C221—P2—C231	83.53 (15)
C211—C212—C213—C214	−0.6 (3)	C226—C221—P2—Cu1	133.86 (14)
C212—C213—C214—F21	−179.82 (17)	C222—C221—P2—Cu1	−40.58 (16)
C212—C213—C214—C215	−0.3 (3)	C236—C231—P2—C211	−94.43 (16)
F21—C214—C215—C216	179.91 (17)	C232—C231—P2—C211	88.54 (14)
C213—C214—C215—C216	0.4 (3)	C236—C231—P2—C221	12.62 (17)
C212—C211—C216—C215	−1.2 (3)	C232—C231—P2—C221	−164.42 (13)
P2—C211—C216—C215	178.61 (15)	C236—C231—P2—Cu1	137.95 (14)
C214—C215—C216—C211	0.4 (3)	C232—C231—P2—Cu1	−39.08 (15)
C226—C221—C222—C223	−2.1 (3)	C322—C321—P3—C311	155.05 (14)
P2—C221—C222—C223	172.58 (15)	C326—C321—P3—C311	−28.92 (16)
C221—C222—C223—C224	−0.5 (3)	C322—C321—P3—C331	−97.54 (14)
C222—C223—C224—F22	−176.99 (17)	C326—C321—P3—C331	78.49 (16)
C222—C223—C224—C225	2.9 (3)	C322—C321—P3—Cu1	28.18 (16)
F22—C224—C225—C226	177.41 (18)	C326—C321—P3—Cu1	−155.80 (12)
C223—C224—C225—C226	−2.5 (3)	C312—C311—P3—C321	135.09 (15)
C224—C225—C226—C221	−0.3 (3)	C316—C311—P3—C321	−49.29 (16)
C222—C221—C226—C225	2.5 (3)	C312—C311—P3—C331	29.31 (16)
P2—C221—C226—C225	−171.92 (16)	C316—C311—P3—C331	−155.07 (14)
C236—C231—C232—C233	−1.5 (3)	C312—C311—P3—Cu1	−94.04 (14)
P2—C231—C232—C233	175.64 (14)	C316—C311—P3—Cu1	81.58 (15)
C231—C232—C233—C234	0.6 (3)	C332—C331—P3—C321	−10.69 (17)
C232—C233—C234—F23	179.96 (16)	C336—C331—P3—C321	164.33 (14)
C232—C233—C234—C235	1.0 (3)	C332—C331—P3—C311	95.75 (16)
F23—C234—C235—C236	179.45 (17)	C336—C331—P3—C311	−89.23 (15)
C233—C234—C235—C236	−1.6 (3)	C332—C331—P3—Cu1	−140.71 (14)
C232—C231—C236—C235	0.9 (3)	C336—C331—P3—Cu1	34.31 (15)
P2—C231—C236—C235	−176.07 (15)	N1—O1—Cu1—P2	−47.17 (15)
C234—C235—C236—C231	0.6 (3)	N1—O1—Cu1—P1	83.31 (15)
C316—C311—C312—C313	1.7 (3)	N1—O1—Cu1—P3	−159.63 (15)
P3—C311—C312—C313	177.38 (14)	C211—P2—Cu1—O1	115.58 (8)
C311—C312—C313—C314	−1.1 (3)	C221—P2—Cu1—O1	−4.47 (8)
C312—C313—C314—F31	179.49 (16)	C231—P2—Cu1—O1	−124.11 (7)
C312—C313—C314—C315	−0.2 (3)	C211—P2—Cu1—P1	−10.33 (7)
F31—C314—C315—C316	−178.93 (17)	C221—P2—Cu1—P1	−130.38 (6)
C313—C314—C315—C316	0.7 (3)	C231—P2—Cu1—P1	109.98 (6)
C314—C315—C316—C311	0.0 (3)	C211—P2—Cu1—P3	−150.88 (7)
C312—C311—C316—C315	−1.2 (3)	C221—P2—Cu1—P3	89.07 (7)
P3—C311—C316—C315	−176.91 (14)	C231—P2—Cu1—P3	−30.58 (6)
C326—C321—C322—C323	−1.5 (3)	C121—P1—Cu1—O1	−3.63 (8)
P3—C321—C322—C323	174.63 (14)	C131—P1—Cu1—O1	−124.75 (7)
C321—C322—C323—C324	0.1 (3)	C111—P1—Cu1—O1	116.10 (7)
C322—C323—C324—F32	−179.21 (16)	C121—P1—Cu1—P2	118.34 (7)
C322—C323—C324—C325	1.5 (3)	C131—P1—Cu1—P2	−2.78 (7)
F32—C324—C325—C326	179.20 (16)	C111—P1—Cu1—P2	−121.93 (6)
C323—C324—C325—C326	−1.5 (3)	C121—P1—Cu1—P3	−102.42 (7)
C324—C325—C326—C321	0.0 (3)	C131—P1—Cu1—P3	136.47 (6)
C322—C321—C326—C325	1.5 (3)	C111—P1—Cu1—P3	17.32 (6)
P3—C321—C326—C325	−174.51 (14)	C321—P3—Cu1—O1	−179.52 (8)
C336—C331—C332—C333	−0.6 (3)	C311—P3—Cu1—O1	58.78 (7)
P3—C331—C332—C333	174.43 (14)	C331—P3—Cu1—O1	−59.34 (7)
C331—C332—C333—C334	1.2 (3)	C321—P3—Cu1—P2	76.24 (7)
C332—C333—C334—F33	−179.35 (16)	C311—P3—Cu1—P2	−45.46 (6)
C332—C333—C334—C335	−0.7 (3)	C331—P3—Cu1—P2	−163.59 (6)
F33—C334—C335—C336	178.36 (15)	C321—P3—Cu1—P1	−65.89 (7)
C333—C334—C335—C336	−0.2 (3)	C311—P3—Cu1—P1	172.40 (6)
C334—C335—C336—C331	0.8 (3)	C331—P3—Cu1—P1	54.28 (6)

### Hydrogen-bond geometry (Å, º)

**Table:**

*D*—H···*A*	*D*—H	H···*A*	*D*···*A*	*D*—H···*A*
C122—H122···O2	0.95	2.30	3.224 (2)	163
C336—H336···O1	0.95	2.17	3.037 (2)	151
C126—H126···F22^i^	0.95	2.40	3.281 (2)	154
C136—H136···O3^ii^	0.95	2.53	3.223 (2)	130
C215—H215···F13^iii^	0.95	2.51	3.301 (2)	141
C315—H315···F33^iv^	0.95	2.50	3.403 (2)	159
C332—H332···F32^v^	0.95	2.48	3.131 (2)	125
C326—H326···F33^vi^	0.95	2.36	3.150 (2)	141

Symmetry codes: (i) *x*−1, *y*+1, *z*; (ii) *x*−1, *y*, *z*; (iii) −*x*+1, −*y*+1, −*z*+1; (iv) *x*, *y*−1, *z*; (v) −*x*+1, −*y*+2, −*z*; (vi) −*x*+2, −*y*+2, −*z*.
